# Identification of the contribution of the ankle and hip joints to multi-segmental balance control

**DOI:** 10.1186/1743-0003-10-23

**Published:** 2013-02-22

**Authors:** Tjitske Anke Boonstra, Alfred C Schouten, Herman van der Kooij

**Affiliations:** 1 Laboratory for Biomechanical Engineering, MIRA institute for biomechanical technology and technical medicine, University of Twente, Faculty of Engineering Technology, PO Box 217, Enschede, AE 7500, The Netherlands; 2 Department of Biomechanical Engineering, Delft University of Technology, Mekelweg 2, Delft, CD, 2628, The Netherlands

**Keywords:** Balance control, Closed-loop system identification, Multivariate systems, Asymmetry

## Abstract

**Background:**

Human stance involves multiple segments, including the legs and trunk, and requires coordinated actions of both. A novel method was developed that reliably estimates the contribution of the left and right leg (i.e., the ankle and hip joints) to the balance control of individual subjects.

**Methods:**

The method was evaluated using simulations of a double-inverted pendulum model and the applicability was demonstrated with an experiment with seven healthy and one Parkinsonian participant. Model simulations indicated that two perturbations are required to reliably estimate the dynamics of a double-inverted pendulum balance control system. In the experiment, two multisine perturbation signals were applied simultaneously. The balance control system dynamic behaviour of the participants was estimated by Frequency Response Functions (FRFs), which relate ankle and hip joint angles to joint torques, using a multivariate closed-loop system identification technique.

**Results:**

In the model simulations, the FRFs were reliably estimated, also in the presence of realistic levels of noise. In the experiment, the participants responded consistently to the perturbations, indicated by low noise-to-signal ratios of the ankle angle (0.24), hip angle (0.28), ankle torque (0.07), and hip torque (0.33). The developed method could detect that the Parkinson patient controlled his balance asymmetrically, that is, the right ankle and hip joints produced more corrective torque.

**Conclusion:**

The method allows for a reliable estimate of the multisegmental feedback mechanism that stabilizes stance, of individual participants and of separate legs.

## Background

Maintaining an upright posture is a relatively easy task for healthy humans [1,2]. However, bipedal upright stance is inherently unstable, as small deviations from the upright posture result in disturbing torques due to gravity, which drives the system further away from upright posture [3]. To stay upright, the body generates corrective torques to counteract the effects of internal (e.g., motor and sensory noise) and external (e.g., uneven surfaces) perturbations.

When postural deviations are small, the body is often simplified as an inverted pendulum pivoting at the ankles, which describes the so-called ankle strategy [3-5]. However, several studies demonstrated that human movement during stance is multi-segmental [6-8] and for example, the hips substantially contribute to upright stance (i.e., the hip strategy [9]). Human balance control is a closed-loop multi-segmental process, i.e., sensory signals about the movement of the body are fed back to the central nervous system (CNS), and the CNS controls the muscles to generate adequate responses [10,11]. In a noisy closed-loop system, like human balance control, causality is difficult to determine and the dynamics of the different components (i.e., the body and the stabilizing mechanisms located in the CNS) affect both the input (joint angles) and output signals (joint torques). To “open” the loop and to separate the dynamics of the different components, the balance system need to be perturbed [11,12]. Furthermore, when estimating the dynamics in a **multivariate** system, **multiple** perturbations need to be applied [13,14].

Most studies investigating the multivariate nature of balance control do not take the multivariate noisy closed-loop nature into account, by either not using perturbations [15-17], or by using only one perturbation [18-20]. Only two studies investigated the multivariate nature of balance control by applying two perturbations [2,21].

Fujisawa and colleagues [21] investigated the role of the hip joint to upright stance by applying pseudorandom perturbations (bandwidth 0 - 0.83 Hz) while manipulating the support surface width. Subsequently, an ARMAX model (with joint angles as input and joint torques as outputs) was fitted to the data to obtain the Frequency Response Functions (FRFs) of a two-segment model of balance control. Results showed an increase of balance contribution of the hip joint, when the support surface became narrower.

Jeka and colleagues [2] identified neural feedback during upright stance in 18 subjects, while applying two mechanical perturbations (springs attached to a linear motor) and one sensory perturbation (visual scene rotations). By comparing the identified neural feedback (from joint angles to weighted electromyograms (EMGs) of the leg and trunk segments) with a large range of cost functions, it was concluded that the CNS stabilizes stance with near minimum muscle activation.

Ageing and many neurological diseases are associated with balance impairments and falls [22]. Understanding the (patho)physiology of upright stance could aid to detect individuals with an increased risk of falls, help to design and evaluate intervention programs or monitor disease progression. Therefore, for clinical applications, it is very important to obtain a reliable individual estimate of balance control.

Of all neurological diseases, PD patients are at the highest risk of falling [22-24], but the pathophysiology of balance impairments in PD remains unclear [25,26]. Recently, it was suggested that one of the factors contributing to decreased balance control in PD patients, is impaired trunk control [27,28] or a decreased intersegmental coordination [29,30]. Another factor could be asymmetrical balance control, that is when one leg produces more force than the other leg to maintain an upright posture. Asymmetries in balance control have been rarely studied in PD, although it is an asymmetrical disease [31]. One study [32] found balance control asymmetries in 24% of the PD participants, indicating that balance asymmetries are important in PD.

Currently, there is no method available that can identify a multisegmental stabilizing mechanism of balance control on an individual level, separating the contribution of the joints of the left and right body side. We developed and evaluated a non-parametric MIMO (Multiple-Input-Multiple-Output) identification method based on the previously used non-parametric system SISO (Single-Input-Single-Output) identification method [4]. To obtain a reliable individual response, periodic perturbations were applied, which have the advantage of having power at specific frequencies, decreasing the measurement time, and increasing the participants response. In addition, the stabilizing mechanisms were estimated based on left and right joint torques (contrary to weighted EMGs; [2]), to be able to investigate balance control asymmetries.

In sum, our goal was to develop a method that can reliably estimate the stabilizing mechanisms of the closed-loop multivariate balance control system of *individual* participants, making a distinction between the contribution of the left and right leg. The (clinical) applicability is demonstrated in an experiment that perturbed the balance of seven healthy participants and a PD patient with a novel device that can apply two independent mechanical perturbations.

## Methods

### Model simulations

A two degree-of-freedom (DoF) balance control model (described extensively in Appendix A) was implemented in Matlab (The Mathworks, Natick, USA) and simulated with Simulink (equations were solved with a fifth order Dormand-Prince algorithm). The human balance model consisted of a two-segment human body with two actuators (ankle and hip), which were controlled using feedback of the joint angles (ankle and hip). In the model, no distinction between the left and right leg was made.

**Figure 1:**
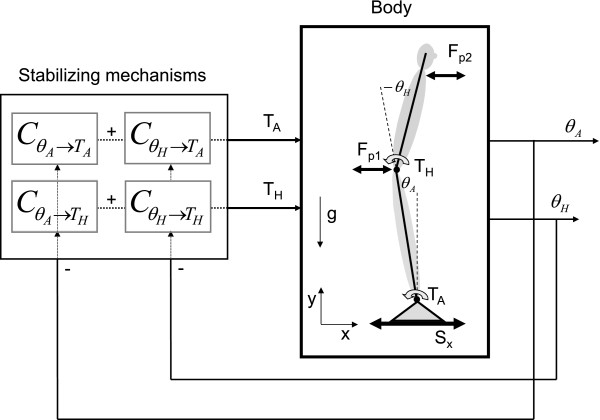
**Multiple-Input-Multiple-Output closed-loop balance control system. **The body mechanics represent the dynamics of a double-inverted pendulum with the corrective ankle and hip torques as inputs and the joint angles as outputs. The stabilizing mechanisms represent the dynamics of the combination of active and passive feedback pathways of the concerned body(part) and generates a torque to correct for the deviation of upright stance. The balance control model can be perturbed with support surface movements (S_x_), perturbation forces at the hip (Fp_1_) or at the shoulder (Fp_2_). Positive torques and positive angles are defined as counterclockwise.

**Figure 2:**
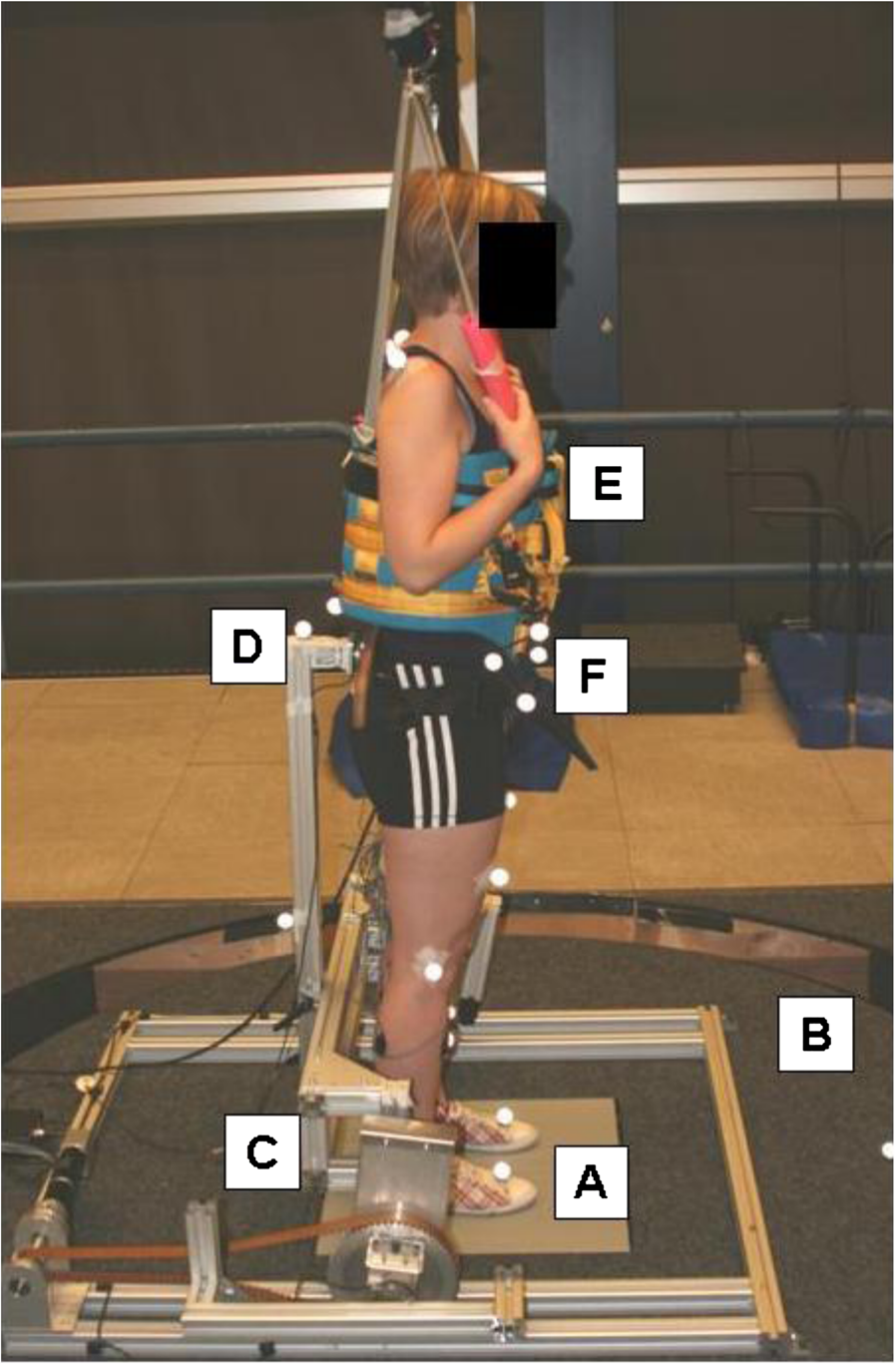
**Experimental set-up. **The participant stands on the dual forceplate (**A**) embedded in the movement platform (**B**). Two independent perturbations are applied with the movement platform (**B**) and the pusher (**C**) in the forward-backward direction. Interaction forces between the pusher (**C**) and the participant are measured with a force sensor (**D**). Actual falls are prevented by the safety harness (**E**). Reflective spherical markers (**F**) measure the movements of the participant.

We perturbed the model with one and two perturbations. Two possible perturbation configurations of the two perturbations were evaluated: 1) external forces at the hip and shoulder (see Figure 1), similar to push-pull rods and 2) a combination of platform forward-backward platform translations and a perturbation torque around the ankle (see Figure 2). Also, simulations without and with pink sensor noise [33] and white (measurement) noise were evaluated:

i. two perturbation forces with noise (2F-N),

ii. two perturbation forces without noise (2F-Nn),

iii. platform perturbation and ankle torque with noise (PLT-N),

iv. platform perturbation and ankle torque without noise (PLT-Nn),

v. one perturbation and one perturbation round with noise (PL-N),

vi. one perturbation and one perturbation round without noise.

The characteristics of the perturbation signals are described in detail in the ‘Disturbance signals’ section and the amplitudes and power spectra are reported in Table 1. The input and output signals of the model were sampled at 120 Hz.

**Table 1:** Perturbation amplitudes, scaling of the perturbation signal, Goodness of Fit and Noise-to-Signal Ratios of the model simulations and the balance control experiment

	**Simulations**						**Experiment**		
	**PLT-Nn**	**PLT-N**	**2F-Nn**	**2F-N**	**PL-Nn**	**PL-N**	**HC**	**HC mean**	**PD patient**
Signal Amplitude	PL: 0.06; T: 20Nm	PL: 0.06m; T: 16Nm	20N	20N	0.12m	0.12m	PL: 0.07; T: 18.4Nm	PL: 0.06m; T: 16Nm	PL: 0.06; T: 20Nm
Scaling	PL: 1/*f*	PL: 1/*f*	-	-	1/*f*	1/*f*	PL: 1/*f*	PL: 1/*f*	PL: 1/*f*
**GOF (1/Hz**^**2**^**)**									
$C_{\theta_{A}\to T_{A}}$	1.11	11.64	1.11	23.64	26.44	29.83	-	-	-
$C_{\theta_{A}\to T_{H}}$	0.25	4.99	0.25	7.16	21.56	22.59	-	-	-
$C_{\theta_{H}\to T_{A}}$	0.79	7.30	0.79	14.42	28.18	36.09	-	-	-
$C_{\theta_{H}\to T_{H}}$	0.25	2.94	0.25	4.38	12.51	14.55	-	-	-
**NSR**									
T_A_	0	0.54	0	0.43	0	1.17	0.55	0.24	0.11
T_H_	0	0.39	0	0.10	0	0.59	0.08	0.28	0.06
_Өsway_	-	-	-	-	-	-	0.10	0.15	0.08

*GOF* goodness of fit, *NSR* noise-to-signal ratio averaged over the two perturbation rounds, *PL* platform, *T* torque. Amplitude values are given in peak-to-peak values. *f* are the frequencies of the perturbation signals.

The GOF is reported for each estimate of the model transfer function $\left(C_{\theta_{A}\to T_{A}},C_{\theta_{A}\to T_{H}},C_{\theta_{H}\to T_{A}}\;\text{and}\;C_{\theta_{H}}{}_{\to T_{H}}\right)$. The median NSR is reported for the input (joint torques) and output (joint angles) for the model simulations and the balance control experiment, averaged over the two perturbation rounds. Six perturbation conditions were simulated: a platform translation+perturbation torque without and with noise (PLT-Nn and PLT-N), two perturbation forces with and without noise (2F-Nn and 2F-N) and a platform translation with and without noise (PL-N and PL-Nn). For the balance control experiment, the median NSRs of one representative healthy control (HC), the average of the healthy controls and of the PD patient are shown.

### Experiment

#### Participants

Seven healthy participants (two female, mean age 65 yrs. std 5.7) and a PD patient (male, 57 yrs) participated in the study. The participants gave written informed consent prior to participation. The protocol was approved by the local medical ethics committee and was in accordance with the Declaration of Helsinki.

#### Apparatus and recording

Two independent perturbations were administered with a computer-controlled six DoF motion platform (Caren, Motek, Amsterdam, The Netherlands) and a custom-built actuated device able to apply perturbing forces in the anterior and posterior direction at the sacrum, called the pusher (see Figure 2). The pusher was attached to the platform and actuated using a series elastic actuator [34] controlled with an electro motor (Maxon motor ag, Sachseln, Switzerland). The pusher was force controlled using a custom-built controller in xPC (The Mathworks, Natick, USA) and had a bandwidth of 10 Hz and a maximum torque of 50 Nm. The gravitational pull due to the weight of the pusher was compensated for, such that the participants did not experience additional forces other than the perturbation force and a small force due to the pusher’s inertia. The interaction force in between the subject and the pusher was measured with a six DoF force transducer (ATI-Mini45-SI-580-20).

Body kinematics and the platform movements were measured using motion capture (Vicon Oxford Metrics, Oxford, UK) at a sample frequency of 120 Hz. Reflective spherical markers were attached to the following anatomical landmarks: the first metatarsal, calcaneus, medial malleolus, the sacrum, the manubrium and the last vertebrae of the cervical spine (C7). In addition, a cluster of three markers was attached to both anterior superior iliac spines on the pelvis. Furthermore, one additional marker was attached to the foot and two markers were attached to the lower leg (one on the tibia) to improve the estimation of the ankle joint rotational axis. Also, markers were attached to the knee (just below the lateral epicondyle) and shoulder joints (just in front of the acromion). Three markers were attached to the platform. Reactive forces from both feet were measured with a dual forceplate (AMTI, Watertown, USA), embedded in the motion platform. The signals from the dual forceplate, the 6 DoF force transducer and the perturbation of the pusher were sampled at 600 Hz and stored for further processing.

#### Procedure

During the experiment, participants stood with their arms folded in front of their chest on the dual forceplate and strapped to the pusher, with a strap band that opened with a click buckle, with their eyes open. They were instructed to maintain their balance without moving their feet, while multisine platform movements and multisine force perturbations were applied simultaneously in the forward-backward direction; see ‘Disturbance signals’. Participants wore a safety harness to prevent falling, but it did not constrain movements, provide support or orientation information in any way.

Before any data was recorded, the participants got acquainted to the perturbations. The experimenter determined the maximal amplitude the participant could withstand while keeping the feet flat on the floor and assessed whether the participant could withstand this amplitude for the total of four trials. Four double perturbation trials of 180s were recorded: in the first two trials, the perturbations had the same sign. In the other two trials, the perturbations had opposite signs. If needed, the participants were allowed rest in between trials.

### Disturbance signals

For both the model simulations and the experiment we used the same perturbation signal. During the model simulations the perturbation signal was used to either produce two perturbing forces, or a combination of a platform translation and a torque around the ankle (see ‘Model simulations’). In the experiment the perturbations were applied with a movement platform and an actuated backboard (see ‘Apparatus and recording’).

The perturbation signal was a multisine with a period of 34.13 s (equal to 2^12^ = 4096 samples at a sample rate of 120 Hz) [4,35]. This signal contained power at 112 frequencies in the range of 0.06–4.25 Hz. To increase the power at the excited frequencies the signal was divided into five frequency bands: 0.06-2.37 Hz (80 frequencies), 2.63-2.84 Hz (8 frequencies), 3.11-3.31 Hz (8 frequencies), 3.57-3.78 Hz (8 frequencies), 4.04-4.25 Hz (8 frequencies). The frequency points outside these frequency bands were not excited. The signal is unpredictable for participants, because the signal consists of many sinusoids. The power of the signal was optimized by crest optimization [13].

### Data analysis

The human body, i.e. the plant, is considered as a double-inverted pendulum, consisting of a leg and a Head-Arms-Trunk (HAT) segment. Stabilizing mechanisms generate ankle and hip torques based on sensory information of the joint angles, (see Figure 1).

The stabilizing mechanisms have passive components such as muscle stiffness, generated by passive muscle properties and tonic activation. The active part incorporates the controller within the CNS (e.g. reflexive muscle activation), muscle activation dynamics, and time-delays, representing the neural signal conduction times.

Movements from the upper body segment will influence the movements of the lower body segment and vice versa due to mechanical coupling [36,37]. The stabilizing mechanisms have to deal with this mechanical coupling, which is especially expressed in coupling terms between ankles and hips (i.e., $C_{\theta_{A}\to T_{H}}\;\text{and}\;C_{\theta_{H}\to T_{A}}$). The direct terms $\left(C_{\theta_{A}\to T_{A}}\;\text{and}\;C_{\theta_{H}\to T_{H}}\right)$ represent the corrective actions of the ankle and hip joint, based on the ankle and hip joint angle. In other words, this system is a multiple input (two joint angles) multiple output (two joint torques) system. When considering the corrective actions of both legs separately, two stabilizing mechanisms are defined; one for each leg [4,38].

For the model simulations, the perturbations, inputs (joint angles) and outputs (joint torques) of the model were determined for further processing. For the experiment these signals were calculated as described below.

From the recorded movement trajectories of the markers, the position of the center-of-mass (CoM) of the predefined segments and of the whole body and the position of the joints were estimated by custom written software [39,40]. In short, in each segment a local coordinate frame was determined on the basis of the position of anatomical landmarks, according to the method described by Koopman et al., 1995 [41]. The mass, CoM position and the inertia tensor moment of the predefined segments (i.e., feet, legs and HAT) and the joint positions were determined with regression equations [41,42]. Subsequently, the CoMs were determined as the weighted sum of the separate segment CoM positions [40]. From the static trial, the average distance in the sagittal plane from the ankle to the total body CoM (i.e., the length of the pendulum; l_CoM_) was determined. The sway angle was calculated from l_CoM_ and the horizontal distance from the CoM to the mean position of the ankles. Forces and torques of the force plate and force sensor were filtered with a fourth-order low-pass Butterworth filter with a cut-off frequency of 8 Hz and subsequently resampled to 120 Hz. Forces and torques of the force plate were corrected for the inertia and mass of the top cover [43]. On the basis of the corrected forces and torques and recorded body kinematics, ankle and hip joint torques were calculated with inverse dynamics [40]. In addition, the applied platform perturbation was reconstructed from the platform markers.

### Multiple-Input-Multiple-Output closed-loop system identification

To obtain a non-parametric spectral estimate of a two DoF multivariate closed-loop system we adopted a method described by Pintelon and colleagues stating that **two** different combinations of a periodic excitation signal, *D(k)*, in **two** separate experiments should be applied [13]. An optimal choice of *D(f)* (maximizing det (**P**(f)) using periodic excitation is given by:

(1) $$P\left(f\right)=\left[\begin{array}{cc} 1 & 1\\ 1 & -1 \end{array}\right]D\left(f\right)$$

With D(*f*) the two perturbation signals. All calculations were performed in the frequency domain with *f* the frequency in Hz. This means that in the first round, both inputs were excited with the same periodic excitation, while in the second round the sign of the second perturbation was changed. These perturbations excited the system and the system responded with movements (joint angles) and torques (corrective joint torques) at the frequencies of the perturbation signal. Corrective torques are the torques that restore the body’s equilibrium in response to motor and sensor noise and the perturbation signal. The estimate from the perturbation to inputs (joint angles) and outputs (corrective joint torques) was first obtained from:

(2) $${\hat{G}}_{\mathrm{py}}\left(f\right)=Y\left(f\right)P^{-1}\left(f\right)$$

With *Y(f),* a two-by-two matrix with on the first column the responses of the first perturbation round (i.e., ankle and hip joint angles or torques) and on the second column the responses of the second perturbation round. ${\hat{G}}_{\mathrm{py}}\left(f\right)$ denotes the estimate of the cross spectral density (CSD) of the disturbance and the outputs (joint angles and joint torques).

Subsequently, the stabilizing mechanisms were estimated using the joint input-output approach [12]:

(3) $${\hat{C}}_{\mathrm{\theta Tc}}\left(f\right)=-{\hat{G}}_{\text{pTc}}\left(f\right){\hat{G}}_{\mathrm{p\theta}}^{-1}\;\left(f\right)$$

With ${\hat{G}}_{\text{pTc}}\left(f\right)$ and ${\hat{G}}_{\mathrm{p\theta}}^{-1}\;\left(f\right)$ the estimated CSD from the perturbations to the corrective torques and from the perturbations to the joint angles*.* Note that C is a two-by-two matrix, see also Figure 1. p is a vector with the two disturbances, θ(*f*) is a vector with ankle and hip joint angles, and T_c_*(f)* is a vector with ankle and hip joint torques for each frequency *f*; all expressed as Fourier coefficients. The method assumes that the system does not change between the two separate perturbation rounds, with the two different combinations of the periodic excitation signal (Eq. 1).

For both the model simulations and the experiment, data was obtained for eight response cycles of the perturbation signal for each perturbation round. Subsequently, the data were Fourier transformed and only the Fourier coefficients at the excited frequencies were used for further processing. These were averaged over the eight cycles, and the average Fourier coefficients were used to calculate the power- and cross spectral density (PSD and CSD, respectively). The PSDs and the CSDs were smoothed by averaging over four adjacent frequency points [44]. The FRFs were calculated according to Eq. 2 and 3 to obtain a non-parametric spectral estimate of the total stabilizing mechanisms.

As the corrective torque which has to be delivered by the participants is dependent on gravity, all FRFs were normalized for the participants’ mass and length, i.e. the gravitational stiffness (*mgl*), with *m* the total body mass, *l* the length of the pendulum (from the ankles to the Center of Mass; CoM), and *g* the gravitational constant. The average FRF over all participants was obtained by taking the mean over the individual normalized FRFs. Note that, as we used a dual forceplate in the experiment, the experimentally obtained Fourier coefficients of the left and right FRFs were added to obtain the total FRFs.

#### Reliability of the estimated MIMO frequency response functions

To determine whether the above described MIMO closed-loop system identification technique gives reliable estimates of the stabilizing mechanisms, several indicators were calculated (described below).

#### Goodness of estimate

For the model simulations, the goodness of fit (GOF) was determined by the object function [33]. This function compared the theoretical Transfer Function (TF) as incorporated in the model (Appendix A) with the estimated FRF as obtained in the model simulations:

(4) $$\text{GOF}={\sum_{f}\left(\frac{\left|H_{\text{theoretical}}\left(f\right)-H_{\text{estimated}}\left(f\right)\right|}{\left|H_{\text{theoretical}}\left(f\right)\right|}\right)}^{2}$$

Where a perfect estimation of the transfer function will result in a GOF of zero, i.e., the lower the GOF the better the estimation.

#### Noise-to-signal ratio

As a result of a periodic perturbation to the balance control system, the system’s response was periodic, while time-variant behavior and/or noise resulted in a stochastic, i.e. non-periodic response [35]. The ratio of the non-periodic (also called the remnant) and periodic response is expressed by the noise-to-signal ratio (NSR):

(5) $$\mathrm{NS}R_{U}\left(f\right)=\frac{\sigma_{u}^{2}\left(f\right)}{\left|U_{p}{\left(f\right)}^{2}\right|}$$

Where *U*_*p*_(*f*) represents the periodic response and *σ*_*u*_^2^(*f*) the variance of the remnant. The NSR was calculated in the frequency domain with *f* frequency in Hz. When multiple realizations are simulated or recorded, the periodic response is obtained by calculating the average over the realizations; the remnant can be estimated by calculating the variance over the realizations.

A small NSR indicates low variability of the system’s response to the perturbation signal over the multiple periods. This indicates time invariant behavior and a low presence of noise [35]. As such, it gives insight into whether linear, time-invariant, system-identification methods can be used and it gives an estimate of how well the system is perturbed. More importantly, it quantifies how reliable the estimate of the stabilizing mechanism is. For example, a NSR of 1 indicates that the recorded data contains 50 percent response and 50 percent remnant. This means that describing the system as a deterministic linear time invariant (LTI) system (expressed by the estimated FRFs in this study) explains 50 percent of the recorded data.

#### Single-Input-Single-Output frequency response functions

To assess whether the two perturbation rounds in the experiment lead to a change in strategy of the participants (i.e., time-variant behavior), we estimated Single-Input-Single-Output (SISO) FRFs from sway angle to ankle torque and from sway angle to hip torque with the joint-input-output-method (Eq. 3; [4]).

#### Coherence

For the SISO FRFs the (magnitude-squared) coherence was calculated between the input signal (perturbations) and output signals (ankle torques, hip torques, and body sway).

(6) $$\gamma_{p,y}^{2}={\left|\phi_{p,y}\right|}^{2}.{\left[\phi_{p,p}\left(f\right).\phi_{y,y}\left(f\right)\right]}^{-1}$$

In which p represents the platform disturbance and y an output signal. *φ*_*p*,*y*_, *φ*_*p*,*p*_ and *φ*_*y*,*y*_ are the CSDs and PSDs of the perturbation and output signals. By definition, coherence varies between 0 and 1, where coherence close to one indicates a low noise level and time-invariant behaviour.

### Balance contribution of the left and right body side

For the experiment we determined the relative contribution of each ankle and hip joint to the total amount of generated corrective torque to resist the perturbations by calculating the contribution of the gain and phase of each leg to the gain and phase of the total body [4]:

(7) $$\text{Contributio}n_{l,r}\left(f\right)=\frac{\mathrm{FR}F_{l,r}\left(f\right)•\mathrm{FR}F_{t}\left(f\right)}{{\left\|\mathrm{FR}F_{t}\left(f\right)\right\|}^{2}}$$

With FRF_l,r_ the left or right FRF and FRF_t_ the total FRF. The ● indicates the dot product of the FRFs. In this way the contribution of the left or right leg to the total balance control was expressed as a proportion. For example, a proportion of 0.8 for the left leg means that the left leg contributed for 80% to the total body stabilization. This was done for each separate MIMO FRF (see Eq. 2 and 3).

## Results

### Model simulations

Table 1 shows the GOF values and NSRs of the different simulations. In case of no sensor and measurement noise, a platform acceleration in combination with a perturbation torque around the ankle (PLT-Nn) gave the same results as two perturbation forces (2F-Nn). In these conditions, the small GOF values indicated that the stabilizing mechanisms were well estimated.

**Figure 3:**
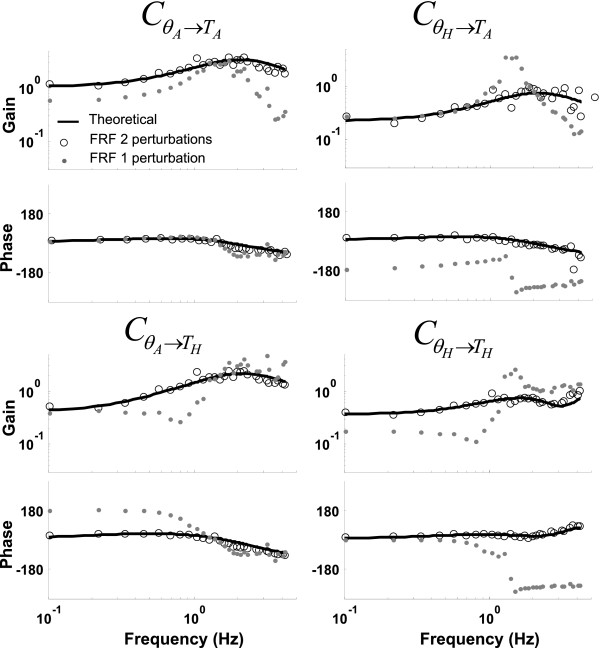
**Theoretical transfer function and estimated frequency response functions **$\left(C_{\theta_{A}\to T_{A}},C_{\theta_{A}\to T_{H}},C_{\theta_{H}\to T_{A}}\;\text{and}\;C_{\theta_{H}\to T_{H}}\right)$**. **Results of the model simulations in the presence of pink and white noise during the condition with a platform acceleration and perturbation torque around the ankle are depicted (PLT-N; open dots) and during the condition with one perturbation (PL-N; solid grey dots). The bold solid line represents the model transfer function of the stabilizing mechanisms. Applying two independent perturbations in combination with a multivariate closed-loop system identification method resulted in a correct estimation of the stabilizing mechanisms (FRF 2 perturbations, indicated with open dots), whereas one perturbation resulted in an erroneous estimate (FRF 1 perturbation, indicated with solid grey dots).

Adding sensor (pink) and measurement (white) noise to the simulations resulted in slightly worse estimations; the GOF values increased. However, the stabilizing mechanisms were still correctly estimated (see Figure 3). Platform acceleration and a perturbation torque around the ankle (PLT-N) resulted in better estimations than two perturbation forces (2F-N).

Figure 3 shows the model transfer function and the estimated frequency response functions of the PLT-N and PL-N conditions. Clearly, applying two perturbations resulted in very good estimations (see also Table 1). However, applying only **one** perturbation to estimate the stabilizing mechanisms of a MIMO system resulted in incorrect estimates, although the responses were time-invariant as shown by the NSRs values. In other words, applying one perturbation resulted in biased estimates of the stabilizing mechanisms. Note that the GOF values of the PL-Nn and PL-N simulations were of the same magnitude.

### Experiment

#### Time series

**Figure 4:**
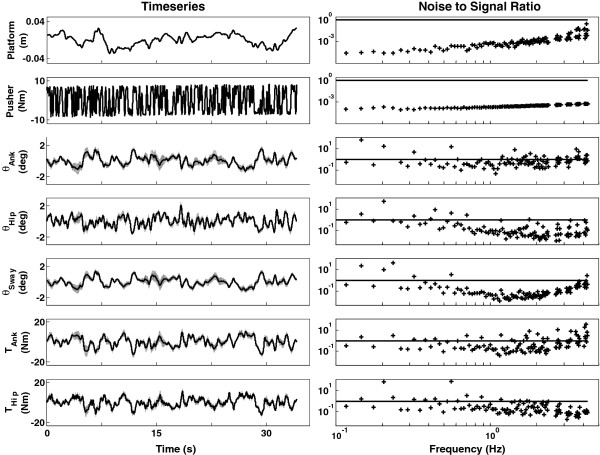
**Timeseries (left panels) and NSRs (right panels) of the first perturbation round of one representative participant. **From top to bottom: platform perturbation, pusher perturbation, ankle angle, hip angle, sway angle, ankle torque, and hip torque. The mean is depicted by the solid line and the standard deviation over the eight cycles by the grey area. The black line in the right panels depicts NSR=1. Ideally, the average NSR of the responses remains below one. The responses of the participant were consistent, as evidenced by small standard deviations over the adjacent segments and low NSRs. This means that a large part of the data is captured by the time-invariant MIMO system identification technique.

Figure 4 shows the perturbations and the response of one representative healthy participant to the applied perturbations. For the healthy controls, the average peak-to-peak amplitudes were 0.068 m (*std*: 0.005) for the platform and 18.4Nm (*std*: 1.05) for the pusher. For the PD patient the peak-to-peak amplitudes were 0.06 m and 20 Nm, see also Table 1. In general, the participant responded in a consistent fashion as indicated by the low standard deviation over the adjacent segments and corresponding low NSRs (Table 1). The average median NSRs (of both perturbation rounds) of all healthy participants were: 0.24 (ankle angle); 0.28 (hip angle); 0.15 (sway angle); 0.56 (ankle torque) and 0.35 (hip torque). Hence, on average, the healthy participants responded in a consistent fashion (see Figure 4). Note, however, that the response of the sway angle was even more consistent than the joint angles. The PD patient was able to complete the balance control experiment without any problems. He could withstand the perturbations and the duration of the trials. The patient also responded in a time-invariant fashion. He had even slightly lower NSRs than the healthy controls.

#### Single-Input-Single-Ouput frequency response functions

**Figure 5:**
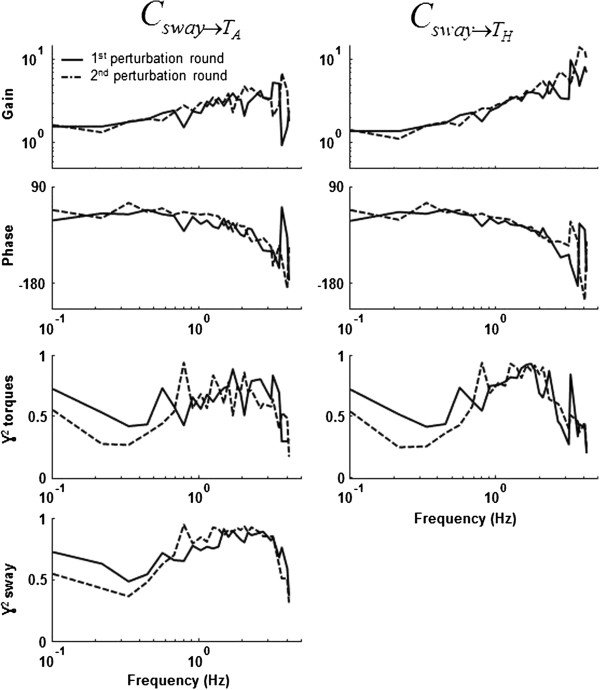
**Single-Input-Single-Output frequency response functions and coherences of first and second perturbation round. **The left panel depicts the FRF from the sway angle to the ankle joint torque; the right panel the FRF from the sway angle to the hip joint torque. The lower panels show the coherence between the perturbation, and the ankle joint torque, hip joint torque, and the sway angle. Similar gains and phases of the frequency response functions of the first and second perturbation round indicate that participants did not change their balance control strategy.

Figure 5 shows the SISO FRFs from sway angle to ankle torque and hip torque of each perturbation round of the healthy controls. Gain and phase of the FRFs and coherence of the joint torques were similar for both perturbation rounds. There was a small discrepancy between the gain of the FRFs at the lower and higher frequencies, but this can be attributed to a less periodic response at these frequencies (indicated by a decreased coherence), and hence the reliability of the FRFs decreased. In addition, the coherence of the sway angle was lower in the second perturbation round, especially at frequencies below 0.7 Hz. This resulted in a slightly worse estimate of the FRFs, compared to the first perturbation round. Similar results were found for the PD patient (data not shown).

#### Multiple-Input-Multiple-Output frequency response functions

**Figure 6:**
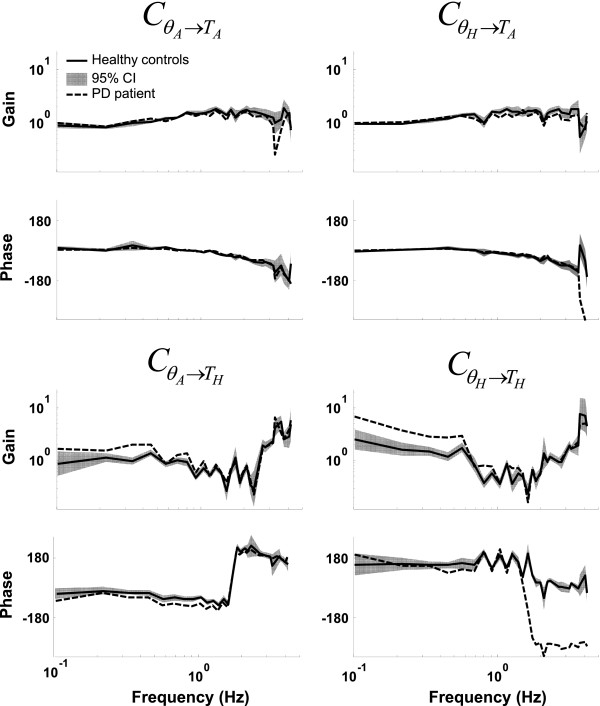
**Multiple-Input-Multiple-Output frequency response functions of the stabilizing mechanisms. **The solid line represents the average of the healthy participants, with the shaded area indicating the 95% confidence interval and the dotted line the Parkinson patient.

The MIMO FRFs of the stabilizing mechanisms of healthy controls and the PD patient are shown in Figure 6. The total of four FRFs are shown: from ankle angle to ankle torque $\left(C_{\theta_{H}\to T_{A}}\right)$, from ankle angle to hip torque $\left(C_{\theta_{A\to }T_{H}}\right)$,from hip angle to ankle torque $\left(C_{\theta_{H\to }T_{A}}\right)$ and from hip angle to hip torque $\left(C_{\theta_{H\to }T_{H}}\right)$ representing the multivariate stabilizing mechanisms of the participants.

In general, for the healthy controls, the relationship between the ankle joint angle and the ankle joint torque remained roughly constant over the whole frequency range, whereas the hip joint gain was high at the low frequencies (<0.7 Hz), low at the mid frequencies (0.7-2 Hz) and increased at higher frequencies (>2 Hz). The coupling between the ankle joint angle to hip torque increased with frequency, whereas the $C_{\theta_{H}\to T_{A}}$ FRF remained roughly constant over the frequency range. The PD patient showed similar gain patterns for the $C_{\theta_{A}\to T_{A}}$ and $\;C_{\theta_{H}\to T_{A}}$ FRFs, but for the $C_{\theta_{A}\to T_{H}}$ and $C_{\theta_{H}\to T_{A}}$ FRFs the gains at the lower frequencies were much higher than those of the healthy controls, indicating an increased postural stiffness in the PD patient.

The phase of the $C_{\theta_{A}\to T_{A}}$ and $C_{\theta_{H}\to T_{A}}$ FRFs of both the HCs and the PD patient decreased with increasing frequency (i.e. a phase lag), indicating a neural time delay. The phase of the $C_{\theta_{A}\to T_{H}}$ showed a phase shift of about +360° around 1.5 Hz. For the phase the largest difference between the HCs and PD patient is found in the $C_{\theta_{A}\to T_{H}}$ FRF. Both groups showed a negative phase shift around 2 Hz, but this shift was larger in the PD patient.

#### Balance contribution of the left and right body side

**Figure 7:**
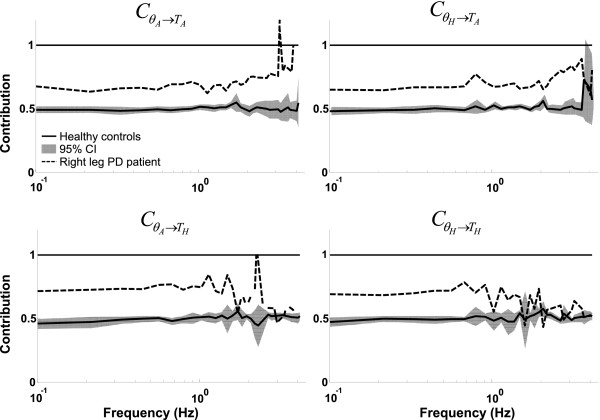
**The average contribution of the right leg of the healthy controls and of the PD patient to each Multiple-Input-Multiple-Output frequency response function. **The average contribution of the right leg for the healthy controls (HC) is shown by the solid line (mean) and the grey area (95% confidence interval). The patient (dashed line) clearly controlled his balance asymmetrically, with the right leg producing more corrective torque than the left leg to resist the perturbations.

In healthy controls both legs contributed equally to the body stabilization; the proportion for each FRF was 0.5. However, for the patient, the right leg contributed more to the balance control than the left leg, and this was the case for both the ankle and the hip joint. Note that the patient is clearly outside the 95% confidence interval (CI) of HCs (see Figure 7). It can be seen that for the FRFs $C_{\theta_{A}\to T_{A}}$ and $C_{\theta_{H}\to T_{A}}$ the proportion between both legs was similar over the whole frequency range, whereas for the FRFs $C_{\theta_{A}\to T_{H}}$ and $C_{\theta_{H}\to T_{H}}$ the asymmetry decreased with increasing frequency and eventually disappeared above 1 Hz.

## Discussion

Bipedal upright stance is multisegmental and requires the coordinated activity of multiple joints, including the ankles and hips. We developed a system identification method to investigate the balance control contribution of the ankles and hips of the left and right leg separately of individual subjects. To investigate both DoF and their interactions in a feedback loop, two perturbations are required. Therefore, in our lab we developed an actuated pusher placed on a motion platform to provide these independent perturbations. Using model simulations, it was demonstrated that these perturbations can identify the system reliably. The (clinical) applicability of the method was demonstrated in seven healthy controls and a PD patient.

### Evaluation of the Multiple-Input-Multiple-Output identification method

#### Model

The model simulations indicated that two independent perturbations are necessary to identify the stabilizing mechanisms of a two DoF MIMO system. Applying only one perturbation to the model resulted in biased estimates of the stabilizing mechanisms. This bias was not influenced by noise level. Two configurations with two independent perturbations were evaluated, that is, a combination of a platform acceleration and a disturbance torque around the ankle and two perturbation forces in the forward-backward direction. The stabilizing mechanism were estimated very well with both configurations, and the differences between the two approaches were small. After adding sensor and measurement noise to the simulation, the stabilizing mechanisms were still estimated well with both configurations, with a slightly worse result when perturbing with two forces. In short, the model simulations showed that the implemented MIMO identification method correctly identified the stabilizing mechanisms indicated by small differences between the theoretical transfer function of the model and the estimated frequency response functions, even in the presence of realistic levels of noise.

#### Experiment

In the experiment with human participants, the perturbations were applied with a motion platform and a custom-made actuated backboard (i.e., the first configuration of the model simulations, which gave slightly better results). The amplitudes of the applied perturbations were easy to withstand, both for the healthy participant as for the PD patient, making the method suitable for use in a large range of participants.

To be clinically relevant, the MIMO identification method should be able to reliably estimate the different segmental contributions to the total balance control of a single participant. First, the quality of our estimation was expressed in the NSR. A low NSR indicates that a large percentage of the data is captured by the estimated stabilizing mechanisms. The NSR gives the ratio between the (periodic) response to the perturbations and the remnant. As the method assumes a LTI system, remnants can be due to a) nonlinearities of the perturbed system, b) time-variant system behavior, c) unmeasured system noise, and d) measurement noise [35]. Note that a low NSR does not necessarily mean that the system is linear.

The average NSRs of the healthy controls of the ankle and hip joint angle and of the ankle and hip torque were 0.24 and 0.28, 0.56 and 0.35, respectively. This results in an average NSR of 0.36, meaning that about 74% (i.e., 1/(1+0.36)) of the response is captured by the estimated stabilizing feedback mechanisms. The PD patient had lower NSRs than the average healthy subject, indicating less variability over the repetitions of the perturbation signal. Unfortunately, it is impossible to compare our results with respect to reliability of the estimates with other studies using two perturbations as these studies did not report any parameters quantifying reliability and only report averages over participants.

Secondly, the used MIMO identification scheme consists of two perturbation rounds: in one round, the perturbations have the same sign, while in the other round, the perturbations have opposite signs. To determine whether participants did not change their balance control behaviour in both perturbation rounds, SISO FRFs for the ankle and hip joints were determined. Gain, phase, and joint torque coherence were comparable for both perturbation rounds; hence the participants did not change their balance control strategies. Note that the sway angle and ankle and hip coherence was lower in the second perturbation round at the lower frequencies (<0.7 Hz), indicating less time invariant behavior (possibly due to fatigue) and/or a higher noise level. This could be due to not randomizing the perturbation rounds. However, this did not result into quantitatively different behavior, as the SISO FRFs were similar for both perturbation rounds.

The last requirement of the method is that it is able to distinguish between the balance contributions of the separate legs to investigate balance asymmetry. By using a dual forceplate and calculating the FRFs for each leg separately, balance control asymmetries were detected in a PD patient.

Perturbing with a different set-up (for example two push-pull rods), at different locations or by using different signals could have elicited different responses. This is not an artifact of the method, but reflects the adaptability of the nervous system [45].

In sum, two unique features of the presented method are the applicability on the individual level and separation of balance contribution of each body side. The first was accomplished by applying multisine perturbation signals, which have the advantage of improving the estimation of FRFs because they concentrate signal power in a limited set of frequencies and are periodic. This results in reliable *individual* results and shorter measurement times [35,46]. Secondly, by measuring the reaction forces of each foot with a dual forceplate balance control, asymmetries can be detected.

### Comparison with other multivariate methods

Most published studies investigating the multivariate nature of balance control do not use perturbations, or use only one perturbation [15,16,18-20]. However, the model simulations indicated that two independent perturbations are required to estimate the stabilizing mechanisms of a multivariate balance control system. Using only one perturbation in the model simulations gave biased and erroneous results.

The other two available methods [2,21] differ from the presented method in this paper. Our method is non-parametric, where Fujisawa [21] used a parametric method (ARMAX model structure). A parametric method has the advantage that it, theoretically, can better separate the measurement noise from the actual signals. However, knowledge about the structure of the system is required. A non-parametric method has the advantage that no prior knowledge of the system is needed [14]. Despite the differences between the applied methods in [21] and in this manuscript the obtained FRFs are in the same range for the low frequencies. That is the $C_{\theta_{A}\to T_{A}}$ FRF starts at unity gain which slightly increases with frequency, whereas the $C_{\theta_{A}\to T_{H}}$_,_$C_{\theta_{H}\to T_{A}}$ and $C_{\theta_{H}\to T_{H}}$ have lower gains at the lower frequencies (~0.05) but also increase with frequency. Note that we can compare the FRFs only in the low frequency range, as Fujisawa and colleagues [21] used a perturbation signal with frequencies up to 0.83 Hz.

Recently, Jeka and colleagues [2,47] used an approach to investigate task goals for upright stance, similar to the one presented here. There are, however, a few fundamental differences. First of all, Kiemel et al. [2] used filtered white noise as a perturbation signal, whereas we used multisines, which have the advantage of improving the individual estimate of the stabilizing mechanisms. Also, they define the input of the stabilizing mechanisms as weighted EMG signals of the anterior and posterior body sides, whereas we have used joint torques. Therefore, the method presented by Jeka and colleagues [2,47] focused on separating the reflexive from the total contribution of the stabilizing mechanisms by measuring EMG signals. This measurement and analysis of EMG signals can easily be added to our method. In order to detect balance control asymmetries, joint torques (which we have used in this study) are more suitable than EMG signals. Differences in EMG amplitude can for example, be due to different electrode placement on contralateral legs, different skin conductivity or due to different background activity, making these signals more prone to measurement artifacts. In addition, joint torques are more suitable for investigating joint stiffness.

### Applications

#### Multisegmental balance control

With a MIMO method and by applying multiple perturbations, multisegmental balance control strategies and the interplay between the joints can be investigated. This approach can be used to test hypotheses about the role of the different joints, and also of sensory information [48].

#### Clinical applications

It has been suggested that PD patients have a decreased intersegmental coordination [29,30] or an increased hip stiffness [25,28]. In this study, the most pronounced difference between the healthy controls and the PD patients was found in the $C_{\theta_{H}\to T_{H}}$ FRF, that is, the PD patient had a higher gain at the lower frequencies, indicating an increased hip stiffness. With our method we can now distinguish between coordination between the upper and lower body and of coordination of the upper/lower body separately. Further investigation of intersegmental coordination could lead to a better understanding of the pathophysiology of balance impairments in PD and possibly improve intervention programs.

The PD patient asymmetrically controlled his balance with both the ankle and the hip joint. This means that one leg contributed more to body stability than the other leg. Balance control asymmetries have been shown before in PD patients, both during quiet stance [32,49] and with a single DoF approach [50], but not taking into account the contribution of the hip joint. The balance asymmetry was also present in the intersegmental coordination, i.e. in the FRFs from ankle angle to hip torque and from hip angle to ankle torque. Hence, our new method has the advantage of assessing balance control asymmetries during perturbations, considering the role of the hip joint and the interplay between joints. This creates the possibility of assessing differences in balance control contribution between distal and proximal joints, and it can be investigated whether balance asymmetries influence intersegmental coordination. Further necessary research in a large group of PD patients and healthy matched controls should demonstrate the (potential) clinical value of the new method.

Furthermore, balance control can also be asymmetrical in stroke patients [4,51]. During the recovering process, restoration of the paretic body side and/or compensation in the non-paretic body side may contribute to improved balance maintenance. Investigating balance control asymmetries provides the possibility of investigating different recovery and compensation strategies during the rehabilitation process.

## Conclusions

We presented a new method to identify the multisegmental stabilizing mechanisms in human stance control using non-parametric system identification techniques and evaluated its performance. Model simulations showed that the newly presented method correctly and reliably estimated the balance control contribution of the ankle and hip joints and interactions between the segments. A balance control experiment showed the application in both healthy and pathological participants. Furthermore, the method can distinguish between the balance control contribution of each ankle and hip joint separately. Taken together, this can be used to create insights into the pathophysiology of postural instability and asymmetry in patients and possibly aid to develop and evaluate treatments.

## Appendix A

### Human balance control model

**Figure 8:**
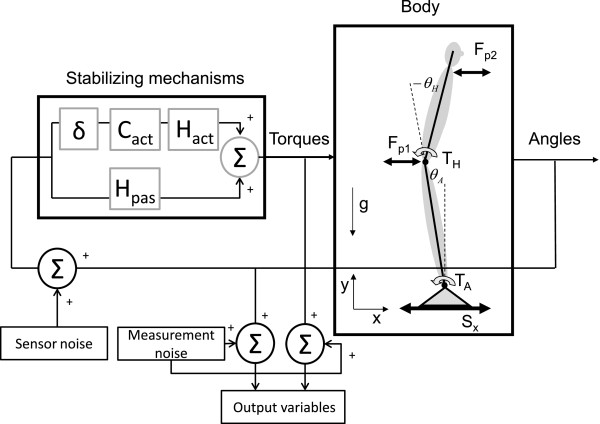
**Model of human balance control. **The model consists of body mechanics and a controller with intrinsic stiffness and damping (C_pas_), an active proportional derivative controller (C_act_), timedelays (δ), muscle activation dynamics (H_act_), sensor and measurement noise. T_ank_ and T_hip _denote the respective joint torques, θ_1 _and θ_2 _the joint angles. S_x _and F_pert _are the force and platform perturbations, respectively. g is the gravitational acceleration.

To test the feasibility of the MIMO identification method, model simulations were performed. A two-DoF mechanical model with a stabilizing mechanism was derived, Figure 8 shows the general model; the derivation of the subsystems is described below.

### Derivation of the equations of motion

Kane’s method (TMT method) was used to derive the equations of motions. In this method, the principles of virtual power are used to rewrite the Newton-Euler equations.

The first step is to define the degrees of freedom (generalized coordinates, q_t_) of the model, which are in this case the support surface (S_x_) and segment angles: θ_1_ and θ_2._

Secondly, a transformation matrix T_j_, which describes the Cartesian coordinates of the center of mass of the segments (x,y coordinates) in the degrees of freedom of the system (i.e., the generalized coordinates S_x_, θ_1_ and θ_2_) is defined:

(8) $$T_{j}=\left[\begin{array}{l} S_{x}\\ S_{x}+d_{1}*\cos\left(\theta_{1}\right)\\ d_{1}*\sin\left(\theta_{1}\right)\\ \theta_{1}\\ S_{x}+l_{1}*\cos\left(\theta_{1}\right)+d_{2}*\cos\left(\theta_{2}\right)\\ l_{1}*\sin\left(\theta_{1}\right)+d_{2}*\sin\left(\theta_{2}\right)\\ \theta_{2} \end{array}\right]$$

With d_1_ and d_2_ the distance to the segment’s center of gravity from the distal point, l_1_ denotes the length of the leg segment. This matrix can be differentiated to the generalized coordinates to obtain the first (T_j,t_) and second derivative (T_j,tl_) of the transformation matrix.

The mass matrix is also defined:

(9) $$M=\text{diag}\left(m_{f},m_{1},m_{1},I_{1},m_{2},m_{2},I_{2}\right)$$

With m_f_, m_1_, m_2_, I_1_ and I_2_, the mass of the foot, the segments masses and inertia’s respectively.

And we also define the external forces and moments (*f*): this is the sum of the gravitational forces F_grav, i_, the external forces F_ext, i ,_ and the joint torques (τ_joint,i_):

(10) $$\begin{array}{ccccccc} f & = & F_{\text{grav},i} & + & F_{\text{ext},i} & + & \tau_{\text{joint},i}\\ \left[\begin{array}{c} F_{\mathrm{sx}}\\ 0\\ -m_{1}*g\\ \left(\tau_{\text{ank}}-\tau_{\text{hip}}\right)+\tau_{p1}\\ 0\\ -m_{2}*g\\ \tau_{\text{hip}} \end{array}\right] & = & \left[\begin{array}{c} 0\\ 0\\ -m_{1}*g\\ 0\\ 0\\ -m_{2}*g\\ 0 \end{array}\right] & + & \left[\begin{array}{c} F_{\mathrm{sx}}\\ 0\\ 0\\ -F_{p1}*p_{1}*\text{sin}\left(\theta_{1}\right)\\ 0\\ 0\\ 0 \end{array}\right] & + & \left[\begin{array}{c} 0\\ 0\\ 0\\ \left(\tau_{\text{ank}}-\tau_{\text{hip}}\right)\\ 0\\ 0\\ \tau_{\text{hip}} \end{array}\right] \end{array}$$

With g the gravitational acceleration (9.81m/s^2^), *τ*_*p*1_ = - *Fp*_1_ * *p*_1_ * sin(*θ*_1_), the perturbation torques due to the external perturbation force. Note that joint moments (τ_ank_ and τ_hip_) are the inputs of the model (forward simulation). Note, that the internal forces are not incorporated in the force matrix; these are implicitly incorporated in the T_j,t_ and T_j,tl_ matrices.

The movement equations with the TMT method lead to the following expression:

(11) $${\bar{M}}_{\text{red}1}\ddot{q}={\bar{f}}_{\text{red}1}$$

With ${\bar{M}}_{\text{red}1}=T^{T}\mathrm{MT}$ and T = (T_i,t_), is the first derivative of the transformation matrix.

(12) $$\text{And}\;{\bar{f}}_{\text{red}1}=T^{T}\left[\sum f-\mathrm{Mg}\right]$$

With f the external forces, M the mass matrix and $g=T_{j,\mathrm{tm}}{\dot{q}}_{t}{\dot{q}}_{m}$; this term corresponds to the centripetal term of the movement equations. In the case of forward simulation, we want to know the accelerations and the torques that are the inputs of the model:

(13) $$\ddot{q}={\bar{M}}^{-1}\bar{f}$$

It can be deduced from Equation 11 and 12 that the equations of motion can be rewritten as:

(14) $$M_{\mathrm{ij}}\left(T_{j,t}\ddot{q}+T_{j,\mathrm{tm}}{\dot{q}}_{t}{\dot{q}}_{m}\right)=F_{\text{grav},i}+F_{\text{ext},i}+\tau_{\text{joint},i}$$

For which we have used the index notation.

### Drivers

In the case of a platform disturbance and a perturbation force, one of the generalized coordinates is specified, namely the platform movement (note that platform accelerations generate the perturbation). Therefore, the platform movement (*S*_*x*_) can be considered as a known degree of freedom, while the remaining degrees of freedom are unknown:

(15) $$q_{t}=\left[\begin{array}{c} q_{u}\\ q_{k} \end{array}\right]$$

This results in a split up of the T_j,t_ matrix in a part for the known (T_j,k_) and unknown degrees of freedom (T_j,u_) and differentiation of these matrices results in $T_{j,\mathrm{kp}}{\dot{q}}_{k}{\dot{q}}_{p}$ and $T_{j,\mathrm{un}}{\dot{q}}_{u}{\dot{q}}_{n}$, respectively. Note that the subscripts u and k denote the unknown and known coordinates, respectively.

By substitution of these matrices in Equation 14, we get:

(16) $$\begin{array}{l} M_{\mathrm{ij}}\left(T_{j,k}{\ddot{q}}_{k}+T_{j,u}{\ddot{q}}_{u}+T_{j,\mathrm{un}}{\dot{q}}_{u}{\dot{q}}_{n}+T_{j,\mathrm{kp}}{\dot{q}}_{k}{\dot{q}}_{p}\right)\\ \;=F_{\text{grav},i}+F_{\text{exter},i}+\tau_{\text{joint},j} \end{array}$$

$$\begin{array}{ll} T_{j,u}^{T}M_{\mathrm{ij}}T_{j,u}{\ddot{q}}_{u}= & T_{j,u}^{T}(F_{\text{grav},i}+F_{\text{ext},i}+\tau_{\text{joint},j}-M_{\mathrm{ij}}T_{j,k}{\ddot{q}}_{k}\\ & -M_{\mathrm{ij}}T_{j,\mathrm{un}}{\dot{q}}_{u}{\dot{q}}_{n}-M_{\mathrm{ij}}T_{j,\mathrm{kp}}{\dot{q}}_{k}{\dot{q}}_{p}) \end{array}$$

$$\begin{array}{l} {\ddot{q}}_{u}=M_{\text{red}1}^{-1}F_{\text{red}1}\\ M_{\text{red}1}=T_{j,u}^{T}M_{\mathrm{ij}}T_{j,u}\\ F_{\text{red}1}=T_{j,u}^{T}(F_{\text{grav},i}+F_{\text{ext},i}+\tau_{\text{joint},j}-M_{\mathrm{ij}}T_{j,k}{\ddot{q}}_{k}\\ \;-M_{\mathrm{ij}}T_{j,\mathrm{un}}{\dot{q}}_{u}{\dot{q}}_{n}-M_{\mathrm{ij}}T_{j,\mathrm{kp}}{\dot{q}}_{k}{\dot{q}}_{p}) \end{array}$$

### Linearization and state space notation

Subsequently, the equations of motion are linearized by differentiating the equations of motion to the states of the system $(\theta_{1}^{0},{\dot{\theta }}_{1}^{0},\theta_{2}^{0},{\dot{\theta }}_{2}^{0}$; the joint angles and joint angle velocity) and to the system inputs and external disturbances $\left(\tau_{\text{ank}},\tau_{\text{hip}},{\ddot{S}}_{x},\right)$ with a first order Taylor approximation:

(17) $$F_{\text{lin}}=f\left(a\right)+f^{\prime }\left(a\right)*\left(x-a\right)$$

Where a is the equilibrium point and x the deviation from the operating point. In our case, the equilibrium position is straight stance (segment angles are 90°, angular velocities, external perturbations, and joint torques are zero). Note that the centripetal term of the movement equations disappears with linearization.

Finally, the equations are rewritten in a state-space notation:

(18) $$\begin{array}{c} \dot{x}=A_{\mathrm{ss}}x+B_{\mathrm{ss}}u\\ y=C_{\mathrm{ss}}x+D_{\mathrm{ss}}u \end{array}$$

With $\dot{x}={\dot{\theta }}_{1},{\ddot{\theta }}_{1},{\dot{\theta }}_{2},{\ddot{\theta }}_{2}$ (thus the derivative of the systems states) and *x* the states. U are the inputs of the system and represent the joint torques, perturbation force, and the platform perturbation.

### Stabilizing mechanism

Passive muscle stiffness and damping were added to each joint; hence, only mono-arcticular muscles are added. The built-up of muscle force is described by the muscle activation dynamics and these were modeled as a second-order dynamical system:

(19) $$H_{\text{act}}\left(\omega \right)=\frac{1}{\frac{1}{\omega_{n}^{2}}s^{2}+\frac{2\beta }{\omega_{n}}s+1}$$

With *s* = jω, the natural frequency (*ω*_n_) was set at 13.8 rad/s (≈2.2 Hz) and the relative damping (*β*) was 0.7. As the sensory signals do not reach the CNS instantaneously (because of neural conduction times) a time delay is also modeled as a pure transport delay, see also Figure 8. Parameter values were taken from the literature [47,52], see Table 2.

**Table 2:** Human balance control model parameters

**Anthropomorphic**		**Controller properties**	
**Parameter**	**Value**	**Parameter**	**Value**
Mass	70 (kg)	Ankle stiffness	293 (Nm/rad)
m_1_	22.54 (kg)	Ankle damping	2.2 (Nm s/rad)
m_2_	47.46 (kg)	Hip stiffness	95 (Nm/rad)
Length	1.70 (m)	Hip damping	27.4 (Nm s/rad)
l_1_	1 (m)	Time delay ankle	60 (ms)
l_2_	0.7 (m)	Time delay hip	40 (ms)
d_1_	0.57 (m)		
d_2_	0.26 (m)		
I_1_	0.65 (m)	*Muscle dynamics*	
I_2_	0.44 (m)	Eigen frequency (ω_n_)	4.4π (rad/s)
g	9.81 (m/s^2^)	Damping (β)	0.7 (-)

Anthropomorphic data is taken from [52]. The controller properties values are taken from [47].

Based on the A and B matrices of the state space equations and intrinsic joint stiffness and damping, the steady-state linear quadratic regulator (LQR), was used to uniquely determine the components of the optimal state feedback matrix C_act_ (see Figure 8). For the optimization, activation dynamics and timedelays are not included in the feedback pathway. The feedback parameters are obtained by minimizing the cost function *J* of the form:

(20) $$J=\int \left(x^{'}\mathrm{Qx}+u^{'}\mathrm{Ru}\right)\mathrm{dt}$$

With *x* the system’s states and *u* the system’s input. Q and R are diagonal matrices; the elements in Q are set to 1 and in R set to 10^6^.

As the sensory signals do not reach the CNS instantaneously (because of neural conduction times) a time delay is included as a pure transport delay, see Figure 8. Parameter values were taken from the literature [6], see Table 2.

Pink sensor noise was created off line, by scaling the power spectrum of a random timeseries by 1.2 such that at 1Hz, the power of the signal was 1.5*10^-7^. Subsequently, this signal was added to the joint angles in the model simulations; this lead to a spontaneous sway of around 0.6° (peak-to-peak amplitude; comparable with human quiet stance data). Measurement noise was modeled as white noise (zero mean and 0.0001 variance) and added to the joint angles and joint torques.

## Abbreviations

CNS: Central nervous system; ARMAX: Auto regressive moving average eXogenous; FRF: Frequency response function; EMG: ElectroMyoGram; PD: Parkinson’s disease; SISO: Single input single output; MIMO: Multiple input multiple output; HAT: Head arms trunk; DoF: Degrees of freedom; CoM: Center of mass; GOF: Goodness of fit; NSR: Noise-to-signal ratio; LTI: Linear time invariant; USA: United States of America; m: Meter; Nm: Newton/m; Hz: Hertz; std: Standard deviation; CLSIT: Closed-loop-system-identification-technique; yrs: Years.

## Competing interests

The authors declare no competing interests.

## Authors’ contributions

TB performed the model simulations, participated in the conception and design of the experiment, performed the data acquisition, analyzed the data, drafted and revised the manuscript. AS has made a substantial contribution drafting and critically revising the manuscript. HvdK conceived of the study and participated in the design and the coordination of the study and critically revised the manuscript. All authors have read and approved the final manuscript.

## Authors’ information

TB received the M.Sc. degree in human movement sciences of the Free University (Amsterdam, The Netherlands) in 2006. She is currently working towards her PhD degree at the laboratory of biomechanical engineering at the University of Twente, in close collaboration with the Radboud University medical Centre in Nijmegen. Her research focuses on developing new techniques and methods based on system identification to investigate and quantify human balance control. Concurrently, she is applying these new and adopted methods for patients with Parkinson’s disease in order to create more insight into the pathophysiology of this disease.

AC received the M.Sc. and Ph.D. degrees in mechanical engineering from the Delft University of Technology, The Netherlands, in 1999 and 2004, respectively. He holds a position as an assistant professor at the Delft University of Technology. He is co-founder of the Delft Laboratory for Neuromuscular Control. His research interest is the field of neuromuscular control and includes techniques to quantify the functional contribution of afferent feedback, neuromuscular modeling, haptic manipulators, and system identification. His research focuses on both able-bodied individuals and individuals suffering from movement disorders.

HvdK (1970, Rotterdam, the Netherlands) received his Phd with honors (cum laude) in 2000 and is professor in Biomechatronics and Rehabilitation Technology at the Department of Biomechanical Engineering at the University of Twente (0.8 fte), and Delft University of Technology (0.2 fte), the Netherlands. His expertise and interests are in the field of human motor control, adaptation, and learning, rehabilitation robots, diagnostic, and assistive robotics, virtual reality, rehabilitation medicine, and neuro computational modeling. Dr.ir. H. van der Kooij has published over 60 papers in the area of biomechatronics and human motor control, and has directed approximately € 6 million in research funding over the past 10 years. He is associate editor of IEEE TBME, member of IEEE EMBS technical committee of Biorobots and was member of several scientific program committees in the field of rehabilitation robotics, bio robotics, and assistive devices. At the UT he is founder and head of Rehabilitation robotics laboratory that developed powered exoskeletons for the rehabilitation of upper and lower extremities. He is founder and head of the Virtual Reality Human performance lab that combines robotic devices, motion capturing and virtual environments to asses and train human balance, walking, and hand-eye coordination.
